# Canine and feline vector-borne diseases of zoonotic concern in Southeast Asia

**DOI:** 10.1016/j.crpvbd.2020.100001

**Published:** 2020-12-11

**Authors:** Viet-Linh Nguyen, Filipe Dantas-Torres, Domenico Otranto

**Affiliations:** a Department of Veterinary Medicine, University of Bari, Bari, Italy; b Department of Immunology, Aggeu Magalhães Institute, Recife, Brazil; c Faculty of Veterinary Sciences, Bu-Ali Sina University, Hamedan, Iran

**Keywords:** Cats, Dogs, Southeast Asia, Vector-borne diseases, Zoonotic, GPELP, Global Programme to Eliminate Lymphatic Filariasis, LF, lymphatic filariasis, MDA, mass drug administration, POC, point-of-care, *s.l.*, *sensu lato*, SEA, Southeast Asia, VBD, vector-borne diseases, VBP, vector-borne pathogens

## Abstract

Dogs and cats are important hosts and reservoirs of many viral, bacterial, protozoal, and helminthic pathogens transmitted by arthropods, including some of zoonotic concern. By sharing the same environment, these companion animals play an important role in the transmission of zoonotic pathogens to humans in various regions and socioeconomic contexts. While canine and feline vector-borne diseases (VBD) are of major concern in wealthy regions (e.g. Europe and North America), less attention has been received in developing countries such as those in Southeast Asia (SEA). This review provides summarized and updated information on canine and feline VBD with emphasis on those of zoonotic concern in SEA. Of these, zoonotic bacteria (i.e. *Bartonella henselae*, *Bartonella clarridgeiae,* and *Rickettsia felis*) and filarial nematodes (i.e. *Brugia malayi*, *Dirofilaria repens,* and *Dirofilaria immitis*) stand out as the most important in veterinary and human medicine. Additionally, the recent finding of *Leishmania infantum* in dogs in SEA raised more concerns about the spreading of this zoonotic agent in this region. Further epidemiological surveys, especially in countries with extremely scant information such as Cambodia, Laos, Myanmar, and Timor-Leste are advocated. Additionally, effective control measures of canine and feline VBD as well as their arthropod vectors should be simultaneously performed for the management of zoonotic infections.

## Highlights

• Many canine and feline vector-borne infections affect animals and humans in Southeast Asia.
• Ticks, fleas and mosquitoes are the most common vectors transmitting pathogens to dogs, cats and humans in Southeast Asia.
• *Bartonella henselae*, *Rickettsia felis* and *Dirofilaria repens* are of concern to human health in this region.
• Collaboration between governments and researchers is encouraged for a better management of vector-borne diseases.

## Graphical abstract

**Figure:**
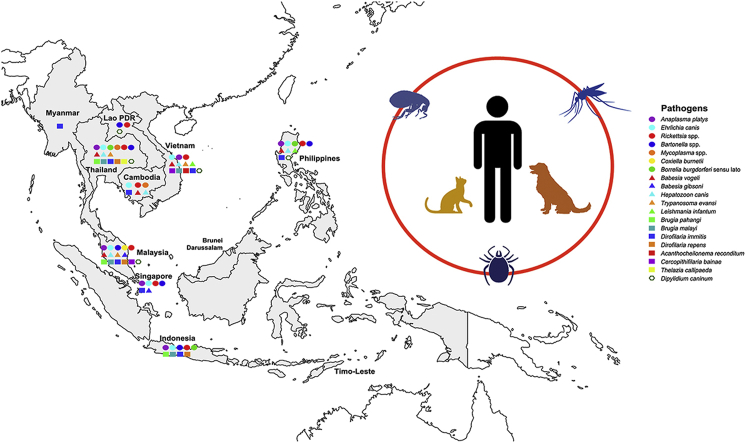

## 1 Introduction

**Fig. 1:**
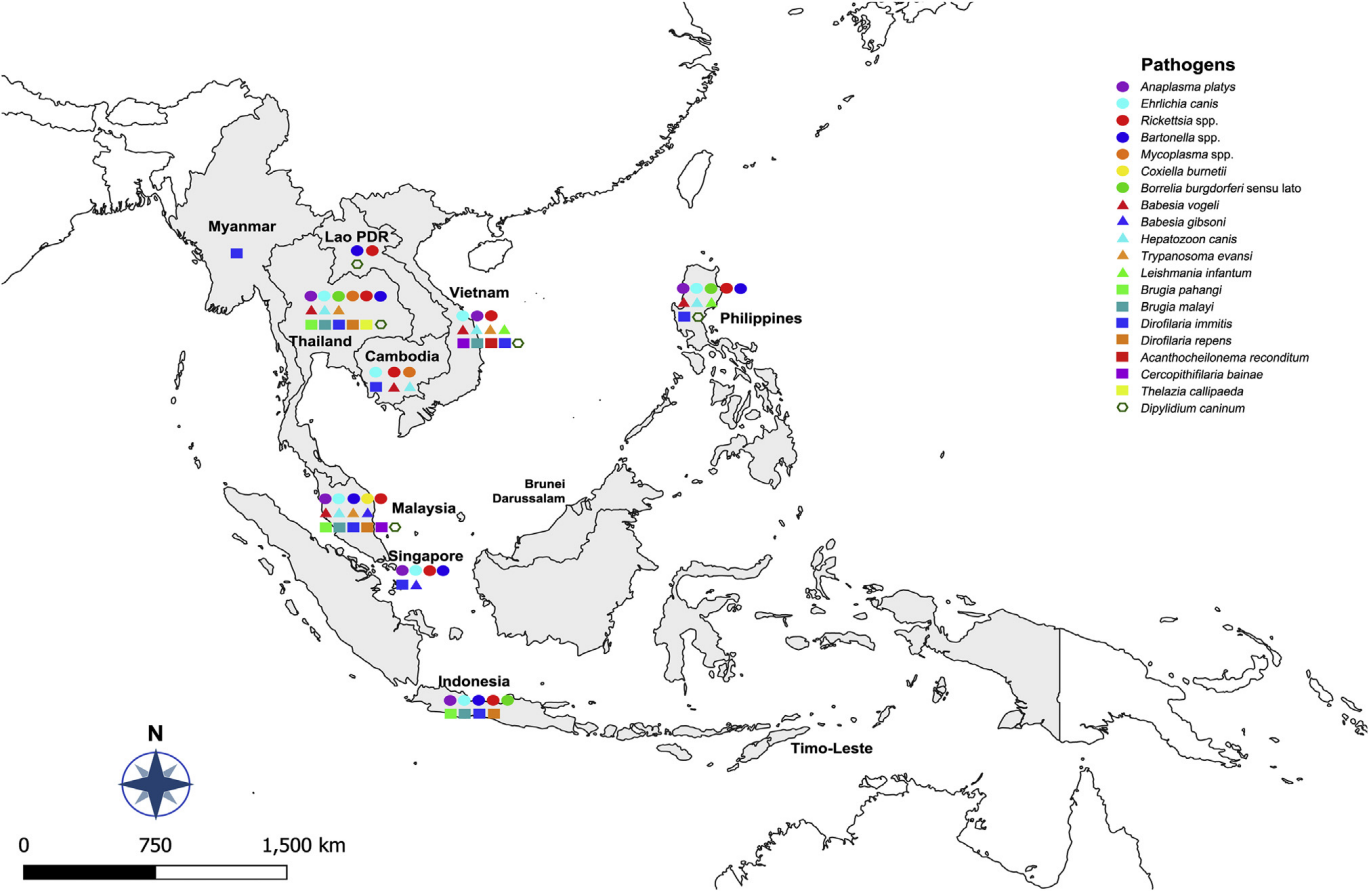
The distribution of vector-borne pathogens in dogs and cats from Southeast Asia.

Southeast Asia (SEA) comprises 11 countries (Brunei Darussalam, Cambodia, Indonesia, Laos, Malaysia, Myanmar, the Philippines, Singapore, Thailand, Timor-Leste, and Vietnam) (Fig. 1), and is home to more than 650 million people with approximately 49% of the population living in rural areas (by 2018) (The World Bank, 2019). In recent decades, SEA countries have been experiencing the fastest-ever economic transformation, which also leads to living standard and health improvements (Fukugawa, 2018). However, this region is still marked by significant social disparities, and remains as a hotspot of many infectious diseases such as dengue fever, malaria, and rabies, which are life-threatening to millions of people (Shepard et al., 2013; Hotez et al., 2015). For example, in spite of the decreasing trend, it is estimated that more than 1,453,000 malaria cases occurred in SEA in 2018, with 2,298 estimated deaths (World Health Organization, 2019).

In addition to the above-mentioned diseases, many zoonotic vector-borne diseases (VBD) have been reported in SEA (Colella et al., 2020; Irwin & Jefferies, 2004; Low et al., 2020), and for some of these dogs and cats play a significant reservoir role. For instance, zoonotic vector-borne pathogens (VBP) such as *Rickettsia felis* and *Bartonella henselae* have been reported in patients having previous contact with dogs, cats and/or ticks and fleas (Edouard et al., 2014; Noopetch et al., 2018). *Dirofilaria immitis*, the causative agent of canine and feline heartworm disease and human pulmonary dirofilariasis (Dantas-Torres & Otranto, 2020), is widely distributed in SEA being reported in dogs from Malaysia, Singapore, and southern Thailand (Colella et al., 2020; Kamyingkird et al., 2017; Lau et al., 2017). Furthermore, although SEA is considered as outside the geographical distribution area of *Leishmania infantum*, the presence of this zoonotic pathogen has been reported in dogs in this region (Colella et al., 2020).

**Fig. 2:**
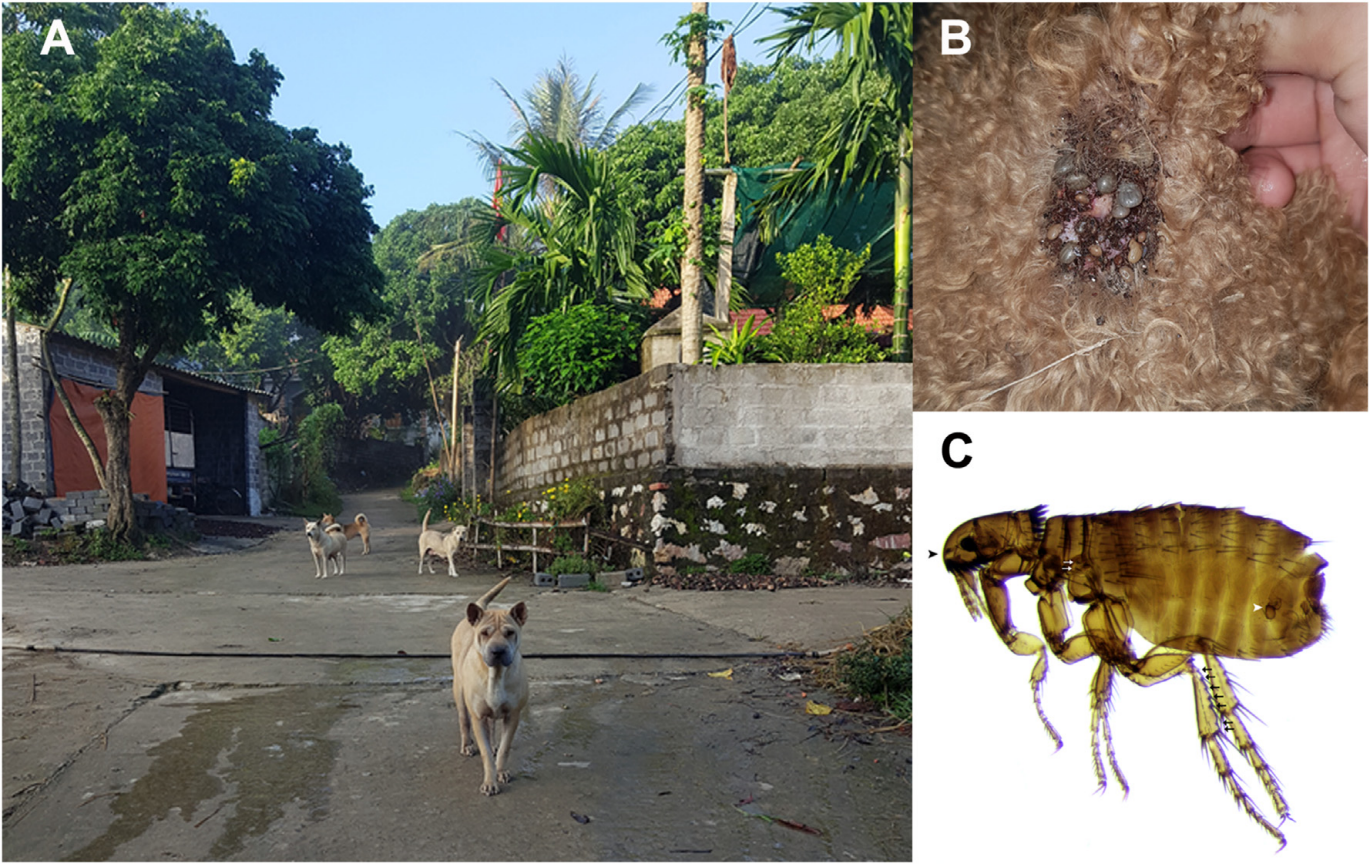
Free-roaming dogs in a rural area of Vietnam (**A**). Heavy infestation of *Rhipicephalus sanguineus* (*sensu lato*) in a pet dog (**B**). A female of *Ctenocephalides orientis* with spermatheca (white arrowhead), strongly rounded anterior margin of the head (arrowhead), 2 setae on the lateral metonotal area (white arrows), and 7 setae-bearing notches on the dorsal margin of the hind tibia (arrows) (**C**).

The awareness regarding the importance of canine and feline VBD is continuously increasing in wealthy regions (e.g. Europe and North America), but less attention has been received in developing regions like SEA. In such regions, there is limited or no access of dogs and cats living in poor suburban or rural areas (Fig. 2A) to veterinary services and preventive measures, thus increasing their risk of acquiring VBP (Dantas-Torres et al., 2020; Otranto et al., 2017). Additionally, the limited availability of financial resources and laboratory facilities in some countries in SEA have historically impaired the scientific knowledge about canine and feline VBD in this region, which certainly has a negative impact on the establishment of appropriate strategies to mitigate zoonotic VBD. Nonetheless, recent multicenter collaborative research has positively impacted on our knowledge on several VBD in SEA, filling some research gaps and uncovering others (Colella et al., 2020). In this perspective, the present article is aimed to provide an update review on VBP and their vectors affecting dogs and cats in SEA, with the main focus on those of zoonotic concern.

## 2 Arthropod vectors of VBP affecting dogs and cats in SEA: what do we know

Ticks, fleas, and mosquitoes represent the most common arthropod vectors transmitting pathogens to dogs and cats in SEA (Irwin & Jefferies, 2004; Colella et al., 2020). Numerous VBP have been detected in dogs and cats as well as in their associated arthropods in this region (Table 1). The brown dog ticks, *Rhipicephalus sanguineus* (*sensu lato*) (Fig. 2B) are the most prevalent ticks found on dogs and, to a lesser extent, cats in SEA (Petney et al., 2019). These ticks act as vectors of many pathogens (e.g. *Anaplasma platys*, *Ehrlichia canis*, *Babesia vogeli,* and *Hepatozoon canis*) affecting dogs, cats, and humans in tropical and subtropical regions (Dantas-Torres & Otranto, 2015). There have been many investigations indicating the presence of two divergent lineages of this tick species (i.e. the tropical and the temperate lineage) in different parts of the world (Dantas-Torres et al., 2013; Nava et al., 2018), and the pathogens transmitted by ticks of these two lineages may also differ (Moraes-Filho et al., 2015). A recent study showed that *R. sanguineus* (*s.l.*) ticks circulating in SEA belong to the tropical lineage (Nguyen et al., 2020b). Additionally, other tick species infesting dogs such as *Rhipicephalus haemaphysaloides*, *Haemaphysalis hystricis*, *Haemaphysalis wellingtoni,* and *Haemaphysalis papuana* have been reported also sporadically (Durden et al., 2008; Kolonin, 1995; Tanskul et al., 1983). However, the role of these ticks as vectors of pathogens in the transmission to dogs in SEA remains unknown.

**Table 1:** Vector-borne pathogens detected in dogs, cats and/or arthropod vectors in Southeast Asia and their zoonotic relevance (i.e. nil, low, moderate, high).

Pathogen	Zoonotic relevance	Isolation source	Country	Reference
**Bacteria**				
*Anaplasma platys*	Low	Dog	Indonesia	Faizal et al. (2019)
			Malaysia	Mokhtar et al. (2013)
			Philippines	Ybañez et al. (2016)
			Singapore	Colella et al. (2020)
			Thailand	Pinyoowong et al. (2008)
			Vietnam	Chien et al. (2019)
		Cat	Thailand	Salakij et al. (2012)
		*Ctenocephalides felis* (from dog)	Laos	Calvani et al. (2020)
		*Rhipicephalus sanguineus* (*s.l.*) (from dogs)	Laos	Nguyen et al. (2020a)
			Malaysia	Low et al. (2018)
			Philippines	Ybañez et al. (2012)
			Thailand	Foongladda et al. (2011)
*Ehrlichia canis*	Low	Dog	Cambodia	Inpankaew et al. (2016)
			Indonesia	Colella et al. (2020)
			Malaysia	Nazari et al. (2013)
			Philippines	Corales et al. (2014)
			Singapore	Colella et al. (2020)
			Thailand	Huggins et al. (2019)
			Vietnam	Colella et al. (2020)
		*R. sanguineus* (*s.l.*) (from dogs)	Malaysia	Low et al. (2018)
			Philippines	Ybañez et al. (2012)
			Thailand	Foongladda et al. (2011)
			Vietnam	Nguyen et al. (2019)
*Rickettsia felis*	High	Dog	Cambodia	Inpankaew et al. (2016)
		*C. felis* (from dogs)	Indonesia	Nguyen et al. (2020b)
			Laos	Kernif et al. (2012)
			Malaysia	Kernif et al. (2012)
			Philippines	Wolf and Reeves (2012)
			Thailand	Nguyen et al. (2020b)
			Vietnam	Nguyen et al. (2020b)
		*C. felis* (from cats)	Laos	Varagnol et al. (2009)
			Indonesia	Nguyen et al. (2020b)
			Philippines	Nguyen et al. (2020b)
			Vietnam	Nguyen et al. (2020b)
		*Ctenocephalides orientis* (from dogs)	Indonesia	Nguyen et al. (2020b)
			Laos	Kernif et al. (2012)
			Malaysia	Kernif et al. (2012)
		*Pulex irritans* (from dog)	Laos	Calvani et al. (2020)
		*R. sanguineus* (*s.l.*) (from dogs)	Philippines	Nguyen et al. (2020b)
		*Heterodoxus spiniger* (from dogs)	Laos	Nguyen et al. (2020a)
*Rickettsia asembonensis*	Nil	*C. felis* (from dogs)	Laos	Nguyen et al. (2020a)
			Philippines	Nguyen et al. (2020b)
		*C. orientis* (from dogs)	Indonesia	Nguyen et al. (2020b)
			Malaysia	Low et al. (2017)
			Philippines	Nguyen et al. (2020b)
			Thailand	Nguyen et al. (2020b)
			Vietnam	Nguyen et al. (2020b)
		*R. sanguineus* (*s.l.*) (from dogs)	Indonesia	Nguyen et al. (2020b)
			Malaysia	Low et al. (2017)
“*Candidatus* Rickettsia senegalensis”	Nil	*C. orientis* (from dogs)	Thailand	Nguyen et al. (2020b)
*Rickettsia* sp. genotype RF2125	Nil	Cat	Thailand	Phoosangwalthong et al. (2018)
		*C. orientis* (from dogs)	Laos	Calvani et al. (2020)
		*Ctenocephalides canis*? (from dogs)	Thailand	Parola et al. (2003b)
*Bartonella henselae*	High	Dog	Philippines	Singer et al. (2020)
		Cat	Indonesia	Marston et al. (1999)
			Malaysia	Hassan et al. (2017)
			Philippines	Chomel et al. (1999)
			Singapore	Nasirudeen and Thong (1999)
			Thailand	Maruyama et al. (2001)
		*C. felis* (from dogs/cats)	Malaysia	Mokhtar and Tay (2011)
		*C. felis* (from cats)	Thailand	Parola et al. (2003b)
		*C. canis*? (from cats)	Thailand	Foongladda et al. (2011)
*Bartonella clarridgeiae*	High	Dog	Thailand	Billeter et al. (2012)
		Cat	Indonesia	Marston et al. (1999)
			Philippines	Chomel et al. (1999)
			Thailand	Maruyama et al. (2001)
		*C. felis* (from dogs)	Laos	Varagnol et al. (2009)
			Thailand	Billeter et al. (2012)
		*C. felis* (from dogs/cats)	Malaysia	Mokhtar and Tay (2011)
		*C. felis* (from cats)	Laos	Calvani et al. (2020)
			Thailand	Parola et al. (2003b)
		*C. orientis* (from dogs)	Laos	Kernif et al. (2012)
		*C. canis*? (from dog)	Thailand	Billeter et al. (2012)
*Bartonella koehlerae*	High	Cat	Thailand	Assarasakorn et al. (2012)
		*C. felis* (from cats)	Thailand	Assarasakorn et al. (2012)
*Bartonella elizabethae*	High	Dog	Thailand	Bai et al. (2010)
*Bartonella quintana*	High	Dog	Thailand	Bai et al. (2010)
*Bartonella rochalimae*	High	*P. irritans* (from dogs)	Laos	Calvani et al. (2020)
		*R. sanguineus* (*s.l.*) (from dogs)	Thailand	Billeter et al. (2012)
*Bartonella vinsonii* subsp. *arupensis*	High	Dog	Thailand	Bai et al. (2010)
*Bartonella vinsonii* subsp. *berkhoffii*	High	Dog	Thailand	Suksawat et al. (2001)
		Cat	Thailand	Srisanyong et al. (2016)
*Bartonella vinsonii* subsp. *vinsonii*	Nil	*C. felis* (from dogs)	Thailand	Billeter et al. (2012)
*Mycoplasma haemofelis*	Nil	Cat	Thailand	Assarasakorn et al. (2012)
		*C. felis* (from cats)	Thailand	Assarasakorn et al. (2012)
“*Candidatus* Mycoplasma haemominutum”	Nil	Dog	Thailand	Liu et al. (2016)
		Cat	Thailand	Assarasakorn et al. (2012)
		*C. felis* (from cats)	Thailand	Assarasakorn et al. (2012)
“*Candidatus* Mycoplasma turicensis”	Nil	Dog	Thailand	Huggins et al. (2019)
		Cat	Thailand	Do et al. (2020)
“*Candidatus* Mycoplasma haematoparvum”	Nil	Dog	Cambodia	Inpankaew et al. (2016)
			Thailand	Kaewmongkol et al. (2017)
*Mycoplasma haemocanis*	Nil	Dog	Cambodia	Inpankaew et al. (2016)
			Thailand	Kaewmongkol et al. (2017)
*Coxiella burnetii*	High	Dog	Malaysia	Tukur et al. (2019)
		*R. sanguineus* (*s.l.*) (from dogs)	Malaysia	Watanabe et al. (2015)
*Borrelia burgdorferi* (*s.l.*)	High	Dog	Indonesia	Colella et al. (2020)
			Philippines	Colella et al. (2020)
			Thailand	Sthitmatee et al. (2016)
**Protozoans**				
*Babesia vogeli*	Nil	Dog	Cambodia	Inpankaew et al. (2016)
			Malaysia	Prakash et al. (2018b)
			Philippines	Galay et al. (2018)
			Thailand	Piratae et al. (2015)
		Cat	Thailand	Simking et al. (2010)
		*R. sanguineus* (*s.l.*) (from dogs)	Malaysia	Prakash et al. (2018b)
			Philippines	Galay et al. (2018)
			Vietnam	Nguyen et al. (2019)
*Babesia gibsoni*	Nil	Dog	Malaysia	Mokhtar et al. (2013)
			Singapore	Colella et al. (2020)
		*R. sanguineus* (*s.l.*) (from dogs)	Malaysia	Prakash et al. (2018b)
*Hepatozoon canis*	Nil	Dog	Cambodia	Inpankaew et al. (2016)
			Malaysia	Prakash et al. (2018a)
			Philippines	Galay et al. (2018)
			Thailand	Piratae et al. (2015)
			Vietnam	Colella et al. (2020)
		Cat	Thailand	Jittapalapong et al. (2006)
		*R. sanguineus* (*s.l.*) (from dogs)	Malaysia	Prakash et al. (2018b)
			Philippines	Galay et al. (2018)
			Thailand	Nguyen et al. (2020b)
			Vietnam	Nguyen et al. (2019)
		*R. sanguineus* (*s.l.*) (from cats)	Philippines	Nguyen et al. (2020b)
*Trypanosoma evansi*	Low	Dog	Malaysia	Rajamanickam et al. (1985)
			Thailand	Barameechaithanun et al. (2009)
			Vietnam	Bui et al. (2020)
*Leishmania infantum*	High	Dog	Philippines	Colella et al. (2020)
			Vietnam	Colella et al. (2020)
**Helminths**				
*Brugia pahangi*	Low	Dog	Malaysia	Colella et al. (2020)
			Thailand	Satjawongvanit et al. (2019)
		Cat	Indonesia	Palmieri et al. (1985)
			Malaysia	Tan et al. (2011)
			Thailand	Nuchprayoon et al. (2006)
*Brugia malayi*	High	Dog	Thailand	Satjawongvanit et al. (2019)
			Vietnam	Colella et al. (2020)
		Cat	Indonesia	Palmieri et al. (1985)
			Malaysia	Al-Abd et al. (2015)
			Thailand	Chansiri et al. (2002)
*Dirofilaria immitis*	High	Dog	Cambodia	Inpankaew et al. (2016)
			Indonesia	Erawan et al. (2018)
			Malaysia	Lau et al. (2017)
			Myanmar	Aung (2014)
			Philippines	Theis et al. (2008)
			Singapore	Colella et al. (2020)
			Thailand	Kamyingkird et al. (2017)
			Vietnam	Colella et al. (2020)
		Cat	Indonesia	Colella et al. (2020)
			Malaysia	Mak et al. (1980)
			Thailand	Kamyingkird et al. (2017)
*Dirofilaria repens*	High	Cat	Indonesia	Palmieri et al. (1985)
			Malaysia	Al-Abd et al. (2015)
			Thailand	Wongkamchai et al. (2014)
*Thelazia callipaeda*	Moderate	Dog	Thailand	Bhaibulaya et al. (1970)
*Acanthocheilonema reconditum*	Nil	Cat	Thailand	Wongkamchai et al. (2014)
*Cercopithifilaria bainae*	Nil	*C. felis* (from cats)	Vietnam	V.-L. Nguyen (unpublished data)
		*R. sanguineus* (*s.l.*) (from dogs)	Malaysia	Latrofa et al. (2014)
*Dipylidium caninum*	Low	Dog	Malaysia	Ngui et al. (2014)
			Philippines	Colella et al. (2020)
			Thailand	Rojekittikhun et al. (2014)
			Vietnam	Nguyen et al. (2015)
		Cat	Laos	Scholz et al. (2003)
			Malaysia	Mohd Zain et al. (2013)
			Thailand	Jittapalapong et al. (2007)
		*C. felis* (from cats)	Malaysia	Low et al. (2017)
		*Felicola subrostratus* (from cats)	Malaysia	Low et al. (2017)

A “?” indicates uncertain data on species identification of *C. canis*.

The cosmopolitan cat flea *Ctenocephalides felis* is commonly found on dogs and cats around the world, including SEA (Lawrence et al., 2019). This flea species is involved in the transmission of many zoonotic bacterial pathogens (e.g. *B. henselae*, *Bartonella clarridgeiae,* and *R. felis*) (Bitam et al., 2010), and also acts as the intermediate host of the tapeworm *Dipylidium caninum* (Guzman, 1984). Whilst the dog flea *Ctenocephalides canis* is climatically restricted to the temperate regions, *Ctenocephalides orientis* (Fig. 2C) is mainly distributed in tropical Asia (i.e. India and SEA) (Colella et al., 2020; Hii et al., 2015; Kernif et al., 2012). Indeed, recently acquired knowledge (Calvani et al., 2020; Colella et al., 2020; Lawrence et al., 2019) indicates that previous reports of *C. canis* parasitizing domestic dogs in SEA probably refer to *C. orientis*, due to their strong morphological similarity. The vector competence of *C. orientis* in transmitting pathogens remains unclear although this flea species has been found to carry some rickettsiae such as *Rickettsia asembonensis* and *Rickettsia* sp. genotype RF2125 (Nguyen et al., 2020; Phoosangwalthong et al., 2018).

Mosquitoes play an important role in the transmission of various pathogens to dogs, cats, and humans worldwide, including SEA. The occurrence of *Dirofilaria* spp. and *Brugia* spp. has been widely reported in this region, and mosquitoes of the genera *Aedes*, *Armigeres,* and *Mansonia* are responsible for the transmission of these filarial pathogens (Denham & McGreevy, 1977; Irwin & Jefferies, 2004).

## 3 Vector-borne pathogens of zoonotic concern

*Rickettsia felis* is an emerging bacterial pathogen, which can be found in mammalian hosts and arthropods worldwide, with *C. felis* acting as the main vector and reservoir for this pathogen (Legendre & Macaluso, 2017; Parola, 2011). More recently, dogs have been demonstrated as competent reservoir hosts of *R. felis* with the infection resulting mostly subclinical symptoms (Ng-Nguyen et al., 2020). Since the first human case of flea-borne spotted fever attributed to *R. felis* in Thai-Myanmar border (Parola et al., 2003a), several cases of *R. felis* infection in patients with non-specific febrile illness have been documented in SEA, including Thailand (Edouard et al., 2014), Laos (Dittrich et al., 2014), Vietnam (Le-Viet et al., 2019), and Indonesia (Mawuntu et al., 2020). This pathogen has been detected in *C. felis* from dogs and cats from Indonesia, Laos, Malaysia, the Philippines, Thailand, and Vietnam (Kernif et al., 2012; Nguyen et al., 2020b). Meanwhile, a study reported that 10.9% of 101 free-roaming owned dogs from Cambodia were molecularly positive for *R. felis* (Inpankaew et al., 2016).

Cats are the main reservoirs for *B. henselae*, *B. clarridgeiae,* and *Bartonella koehlerae*, which cause cat scratch disease and endocarditis in humans (Chomel et al., 2006). Of these, *B. henselae* and *B. clarridgeiae* were reported in cats and their fleas from Indonesia, Malaysia, the Philippines, Singapore, and Thailand with prevalences of up to 60% (Chomel et al., 1999; Marston et al., 1999; Maruyama et al., 2001; Mokhtar & Tay, 2011; Nasirudeen & Thong, 1999), whereas *B. koehlerae* was detected for the first time in SEA in cats and *C. felis* from Thailand (Assarasakorn et al., 2012). Human infection by *B. henselae* has been reported to cause endocarditis in Laos and Thailand (Noopetch et al., 2018; Rattanavong et al., 2014; Watt et al., 2014), and ocular neuroretinitis in Malaysia (Tan et al., 2017). Additionally, *Bartonella vinsonii* subsp. *berkhoffii*, another important agent of human endocarditis, was detected in cats and dogs from Thailand (Srisanyong et al., 2016; Suksawat et al., 2001). Several other zoonotic species and subspecies of *Bartonella* have been identified in dogs from Thailand including *B. clarridgeiae*, *Bartonella elizabethae*, *Bartonella quintana,* and *B. vinsonii* subsp. *arupensis* (Bai et al., 2010; Billeter et al., 2012), with the latter being also found in Thai patients (Bai et al., 2012). Recently, *B. henselae* was also found to infect dogs in the Philippines (Singer et al., 2020).

Lyme borreliosis by *Borrelia burgdorferi* (*s.l.*) is mostly prevalent in the temperate northern hemisphere (Lantos et al., 2014). These bacteria are transmitted to dogs and humans by tick species of the genus *Ixodes*, particularly *Ixodes ricinus* and *Ixodes persulcatus* in Europe and northern Asia, and *Ixodes scapularis* in North America (Dantas-Torres et al., 2012a; Jongejan & Uilenberg, 2004). The presence of *B. burgdorferi* (*s.l.*) has also been serologically and molecularly confirmed in dogs in Thailand (Sthitmatee et al., 2016). Recently, *B. burgdorferi* (*s.l.*) has been serologically diagnosed in dogs from Indonesia and the Philippines (Colella et al., 2020). Some unexplained seropositive results have also been reported in other non-endemic areas worldwide, which could also suggest the occurrence of rare, but possible cross-reaction (Azzag et al., 2015; Maggi & Krämer, 2019).

*Leishmania infantum*, the causative agent of canine leishmaniasis, is among the most important zoonotic VBP of dogs, which has been found in all continents, except Oceania (Dantas-Torres et al., 2012b). In the Old World, this parasite is transmitted by various species of phlebotomine sand flies within the genus *Phlebotomus* (Killick-Kendrick, 1990), and causes visceral and/or cutaneous leishmaniasis in dogs and humans (Dantas-Torres et al., 2012b). Canine leishmaniasis is endemic in many regions of the world, such as South America and the Mediterranean basin (Otranto & Dantas-Torres, 2013). In SEA, *L. infantum* has been serologically diagnosed in dogs in the Philippines and Vietnam (Colella et al., 2020), and the presence of *L. infantum* has also been molecularly confirmed in one patient in Thailand (Maharom et al., 2008). Even though SEA is not considered as a *L. infantum*-endemic area, a study showed a high seroprevalence (55.3%) in immigrant workers in Malaysia; however, none of the tested phlebotomine sand flies from the same survey was found positive for *Leishmania* spp. by PCR (Noor Azian et al., 2016), which raises some doubts about the origin of these infections.

*Trypanosoma evansi* is a protozoan transmitted by hematophagous flies of the genera *Stomoxys* and *Tabanus*, which has been found in various mammalian hosts including bovines, rodents, canines, and humans in tropical and subtropical regions (Aregawi et al., 2019). This parasite is of great veterinary concern due to its ability to cause severe illness in animals such as dogs and horses (Desquesnes et al., 2013). Many cases of canine trypanosomiasis have been reported in South America, Africa, Europe, and Asia (Defontis et al., 2012; Howes et al., 2011; Panigrahi et al., 2015; Rashid et al., 2014; Rjeibi et al., 2015). In SEA, some cases of canine trypanosomiasis have been reported in Malaysia and Thailand (Barameechaithanun et al., 2009; Rajamanickam et al., 1985). The presence of *T. evansi* has also been molecularly detected in a dog in Vietnam (Bui et al., 2020). Other than dogs, *T. evansi* is highly prevalent in cattle and water buffaloes in SEA (Desquesnes et al., 2009; Verloo et al., 2000). Notably, some cases of human infections with *T. evansi* have been reported, including one from Vietnam (Joshi et al., 2006; Powar et al., 2006; Van Vinh Chau et al., 2016), raising concerns about its zoonotic potential in endemic regions.

Lymphatic filariasis (LF), commonly known as elephantiasis, is one of the neglected tropical diseases, and it has been considered for a long time as endemic in SEA (Noordin et al., 2013). An estimated 15 million people in SEA are affected by LF (Sudomo et al., 2010). The disease is mainly due to the infection with *Wuchereria bancrofti*, *Brugia malayi,* and *Brugia timori*, which are transmitted by mosquitoes (World Health Organization, 2010). In particular, *W*. *bancrofti* is responsible for 90% LF cases, and the remaining are mostly due to *B. malayi* (World Health Organization, 2010). Domestic cats are recognized as reservoirs of the zoonotic nocturnal subperiodic form of *B. malayi*. The infection has been reported in cats with a prevalence ranging from 8.2% in Malaysia (Al-Abd et al., 2015) up to 28.3% in southern Thailand (Chansiri et al., 2002). The recent findings of *B. malayi* DNA in dogs from Thailand and Vietnam (Colella et al., 2020), and from other countries (e.g. Sri Lanka and India) in neighboring regions (Mallawarachchi et al., 2018; Manoj et al., 2020) highlight the importance of domestic dogs as potential reservoirs for this zoonotic filarial nematode in these countries. *Brugia malayi* is one of the main targets of the Global Programme for the Eliminate of Lymphatic Filariasis (GPELF), which aimed to eradicate this disease as a public health problem by 2020 through mass drug administration (MDA). Some countries such as Cambodia, Thailand, and Vietnam have already achieved the eradication of LF and others are still accomplishing this goal (World Health Organization, 2020). Mosquitoes of the genus *Mansonia* (e.g. *Mansonia bonneae* and *Mansonia dives*), which have a wide distribution in SEA, are recognized as the main vectors of this filarial nematode (Zielke et al., 1993). *Brugia pahangi*, a species closely related to *B. malayi*, was found in dogs and cats from Indonesia, Malaysia, and Thailand. Although this filarial nematode was not considered as infecting humans under natural conditions, the first cases of human filariasis caused by *B. pahangi* have been reported in Malaysia (Tan et al., 2011). Thereafter, *B. pahangi* was found causing ocular infection in a Malaysian patient, and the microfilariae of this filarial nematode were also found in her cat and in *Armigeres subalbatus* mosquitoes from surrounding areas (Muslim et al., 2013a). This mosquito species has been proven as vector of *B. pahangi* (Muslim et al., 2013b) along with *Mansonia annulata* and *M. dives* (Laing et al., 1960).

Other mosquito-borne filarial nematodes of zoonotic concern in SEA are *D. immitis* and *Dirofilaria repens*. These parasites are widely distributed, and can be found in many animal species, including humans (Dantas-Torres & Otranto, 2020; Otranto et al., 2013; Simón et al., 2012). Approximately, 70 mosquito species mainly from the genera *Culex*, *Aedes,* and *Anopheles* are considered as competent vectors of *D. immitis* and *D. repens*, causing animal and human heartworm and subcutaneous diseases, respectively (Eldridge & Edman, 2000). Among the mosquito vectors, *Aedes aegypti*, *Aedes albopictus,* and *Culex quinquefasciatus*, which are commonly found in rural and urban areas of SEA, may be responsible for the transmission of these two *Dirofilaria* spp. (Tiawsirisup & Kaewthamasorn, 2007; Tiawsirisup & Nithiuthai, 2006). *Dirofilaria immitis* was reported in a human patient in Thailand with a pulmonary nodule at the right lower lobe (Sukpanichnant et al., 1998). Canine heartworm infection caused by *D. immitis* is endemic in SEA, where the prevalence reported in dogs ranges from 16% in Cambodia to 24% in Thailand (Inpankaew et al., 2016; Kamyingkird et al., 2017). Studies also reported the presence of *D. immitis* in cats in Indonesia, Malaysia, and Thailand with the higher infection prevalence (36%) recorded in southern Thailand (Kamyingkird et al., 2017). On the other hand, *D. repens* was also found in cats from those aforementioned countries with the highest prevalence of 12% in Malaysia (Al-Abd et al., 2015). This filarial nematode is endemic in dogs from Europe, where many cases of human subcutaneous/ocular infection have been diagnosed (Capelli et al., 2018; Otranto & Eberhard, 2011). In SEA, many cases of ocular infections with *D. repens* have been reported, including two cases in Thailand (Jariya & Sucharit, 1983; Pradatsundarasar, 1985), four in Malaysia (Rohela et al., 2009), and 11 in Vietnam (Dang et al., 2010; Van De et al., 2012). Additionally, a rare case of subcutaneous infection with *D. repens* on the posterior thoracic region was also reported in Vietnam (Le et al., 2015). Recently, a case of a Thai patient with subconjunctival dirofilariasis caused by *Dirofilaria* sp., closely related to the *Dirofilaria* sp. found in humans in Hongkong and India (To et al., 2012), has been documented (Sukudom et al., 2018). Moreover, two species/genotypes of *D. repens*-like filarial nematodes (referred to as *Dirofilaria* sp. “Thailand II” and *Dirofilaria* sp. “Thailand III”) were reported in cats from Thailand (Yilmaz et al., 2016, 2019), which indicates that the genetic diversity of filarial nematodes in SEA may be currently underestimated.

*Thelazia callipaeda*, also known as the “oriental eye-worm” was initially described in the former Soviet Union and in many countries in the Far East (Otranto et al., 2020). Zoophilic fruit flies of the genus *Phortica* act as intermediate hosts of this parasite in Europe (i.e. *Phortica variegata*) and in SEA (i.e. *Phortica okadai*) (Otranto et al., 2004). This nematode causes ocular infection in carnivores (e.g. dogs, cats, and foxes) and humans (Otranto et al., 2004). Human ocular infections have been reported in Indonesia, Thailand, and Vietnam (Bhaibulaya et al., 1970; Kosin et al., 1989; Van De et al., 2012; Viriyavejakul et al., 2012; Yospaiboon et al., 1989). The infection was also reported in dogs from Thailand (Bhaibulaya et al., 1970). However, none of the dogs and cats tested in a recent survey from SEA countries was found positive for *T. callipaeda* (Colella et al., 2020).

*Dipylidium caninum* is a tapeworm infecting dogs and cats worldwide, with their fleas (e.g. *C. felis*, *C. canis,* and *C. orientis*) and lice (e.g. *Trichodectes canis*) serving as intermediate hosts (Labuschagne et al., 2018). The infection in dogs and cats occurs by accidental ingestion of infected intermediate hosts (Guzman, 1984). Similarly, the infection in humans may occur through this route (García-Agudo et al., 2014; Sapp & Bradbury, 2020), and it has been recorded in at least 24 countries (Jiang et al., 2017). In SEA, since a human case was documented in an old study from the Philippines (Mendoza-Guazon & Abad, 1916), no other case has been reported, although some infections have been detected in neighboring countries such as India (Narasimham et al., 2013) and China (Jiang et al., 2017). The true incidence of *D. caninum* in humans seems to be underestimated considering the asymptomatic infection, and that examination for the presence of proglottids in faeces is seldom performed (Sapp & Bradbury, 2020). Conversely, the infection is commonly found in dogs and cats, especially in stray populations or those from rural areas. Some studies in Malaysia using fecal examination revealed a prevalence of *D. caninum* infection in rural dogs and stray cats reaching up 3.7% and 11.6%, respectively (Mohd Zain et al., 2013; Ngui et al., 2014). Interestingly, a recent molecular characterization of *D. caninum* confirmed the existence of two distinct genotypes (i.e. canine and feline genotypes), which are apparently host specific (Beugnet et al., 2018; Labuschagne et al., 2018). Additionally, *C. felis* and *Felicola subrostratus* collected from cats in Malaysia were also found to harbour DNA of the feline genotype of *D. caninum* (Labuschagne et al., 2018; Low et al., 2017).

## 4 Other VBP affecting dogs and cats

Other VBP of veterinary concern, which affect animal health and welfare, have also been reported in SEA. Some of them may also cause mortality in dogs as it is the case of *E. canis*, the causative agent of canine monocytic ehrlichiosis. This bacterium is widespread and considered as highly virulent to dogs in SEA (Colella et al., 2020; Niwetpathomwat et al., 2006). In the past, this tick-borne pathogen was responsible for the death of hundreds of US military dogs serving in Vietnam (Kelch, 1984). In a study conducted in Thailand, 33% of dogs infected by *E. canis* had fever and in 55% of them the body temperature higher than 40 °C (Niwetpathomwat et al., 2006). Another bacterial pathogen commonly reported in dogs in SEA is *A. platys*, the causative agent of canine cyclic thrombocytopenia, with a prevalence reaching up to 38.5% in stray dogs from Malaysia (Mohammed et al., 2017). This bacterium was also reported in a domestic cat from Thailand (Salakij et al., 2012).

Two species of *Babesia* (*B. vogeli* and *Babesia gibsoni*) have been reported in dogs from SEA (Inpankaew et al., 2016; Prakash et al., 2018b). These parasites may cause clinical conditions (e.g. lethargy, anemia, and thrombocytopenia) as reported for *B. vogeli* in dogs from the Philippines (Ybañez et al., 2017). Another protozoan commonly found in SEA is *H. canis* (Colella et al., 2020; Inpankaew et al., 2016), which is considered less virulent, although it can cause a range of clinical signs in dogs (Baneth et al., 2003).

Haemotropic mycoplasmas, also known as haemoplasmas, are widespread bacteria of cats and other carnivores that can be found all over the world (Barrs et al., 2010; Latrofa et al., 2020; Soto et al., 2017). The infection can induce clinical spectrum ranges from subclinical to haemolytic anemia and life-threatening conditions, particularly in immunocompromised hosts (Willi et al., 2007). Three haemoplasmas (i.e. *Mycoplasma haemofelis*, “*Candidatus* Mycoplasma haemominutum”, and “*Candidatus* Mycoplasma turicensis”) are mostly found infecting cats (Barker, 2019; Do et al., 2020), whereas *Mycoplasma haemocanis* and “*Candidatus* Mycoplasma haematoparvum” are commonly found in dogs (Huggins et al., 2019; Soto et al., 2017). The natural transmission routes of haemoplasmas in dogs and cats remain unclear, though the vector competence of *R. sanguineus* (*s.l.*) and *C. felis* to transmit *M. haemocanis* and *M. haemofelis*, respectively, has been considered (Senevtratna et al., 1973; Woods et al., 2005). Direct transmission by biting and blood transfusion have also been reported (Willi et al., 2007). In SEA, the infection with *Mycoplasma* spp. has received less attention, with few studies conducted so far. However, high prevalence of *Mycoplasma* spp. was reported in community dogs (40%) and cats (38%) from Thailand (Do et al., 2020; Huggins et al., 2019) as well as in free-roaming dogs (12.8%) from Cambodia (Inpankaew et al., 2016), suggesting the common occurrence of these bacteria in dogs and cats in SEA.

## 5 Managing zoonotic VBP

### 5.1 Diagnosis

Several limitations (e.g. relatively high costs of diagnostic tests, scarce laboratory infrastructures, and lack of diagnostic expertise) affect the ability of veterinarians to achieve reliable diagnoses of VBP of dogs and cats in SEA as well as other developing regions (Dantas-Torres et al., 2020; Otranto, 2015). Due to its relatively low cost and high sensitivity, the Knott's test is the most popular procedure for detecting microfilariae in SEA (Noordin et al., 2013). Although the presence of microfilariae can be microscopically identified in the bloodstream of infected animals (Panarese et al., 2020), their identification based on morphology and measurements may be troublesome. For example, microfilariae of *B. malayi* are around 220 μm in length and 5 μm in diameter, and those of *B. pahangi* are around 280 × 5 μm (Schacher, 1962; Taylor, 1960). *Dirofilaria immitis* microfilariae are 290–330 μm in length and 5–7 μm in diameter, whereas those of *D. repens* are slightly longer and wider (350–385 × 7–8 μm) (Simón et al., 2012). Moreover, false negative results may occur in cases of adult single-sex infection or low microfilaremia. Many studies have been conducted based on serological surveys by using point-of-care (POC) commercial kits to detect antigen of *D. immitis* females in serum or blood samples (Chelliah & Šlapeta, 2019; Colella et al., 2020; Sukhumavasi et al., 2012; Theis et al., 2008). However, no similar serological tests are available for the diagnosis of other filarial infections. Serological tests such as immunofluorescence antibody, western immunoblot, POC tests (e.g. SNAP 4Dx Plus and SNAP *Leishmania*) have also been applied to detect the presence of antibodies to several pathogens (i.e. *Anaplasma* spp., *Ehrlichia* spp., *Bartonella* spp., *B. burgdorferi,* and *Leishmania* spp.) (Chomel et al., 1999; Colella et al., 2020; Suksawat et al., 2001). Additionally, the misinterpretation of cytology may also lead to the misdiagnosis of some microorganisms, e.g. reports of “*Babesia canis*” in dogs in SEA probably refer to *B. vogeli* (Petney et al., 2019). Therefore, the use of molecular assays for pathogen detection is recommended, as they are faster and more accurate. A study in Thailand revealed a significantly higher prevalence of *D. immitis* infection in dogs and cats by using conventional PCR (cPCR) compared to microscopic examination (Kamyingkird et al., 2017). A real-time fluorescence resonance energy transfer PCR assay has also been used to diagnose the infection with *B. malayi* and *B. pahangi* (Thanchomnang et al., 2010). More recently, a real-time PCR followed by high resolution melting analysis has been developed for the detection of multiple filarial nematodes (i.e. *Acanthocheilonema reconditum*, *B. malayi*, *B. pahangi*, *D. immitis,* and *D. repens*) in cats (Wongkamchai et al., 2014) and also in mosquito vectors (Thanchomnang et al., 2013). Many molecular surveys using cPCR and real-time PCR to detect the presence of bacterial and protozoal pathogens in dogs, cats, and their associated arthropods have been conducted (Colella et al., 2020; Inpankaew et al., 2016; Kernif et al., 2012; Singer et al., 2020). Recently, next-generation sequencing, which provides a powerful tool to diagnose multiple pathogens with higher sensitivity compared to cPCR, has also been applied for the detection of canine VBP in Thailand (Huggins et al., 2019). However, considering the highly required laboratory infrastructures, expertise, and cost per sample tested for molecular assays (Momčilović et al., 2019), the application of molecular techniques for diagnosing VBP in dogs and cats as well as their associated vectors has been limited in some countries such as Cambodia, Laos, Myanmar, and Timor-Leste. As such, the prevalence and clinical importance of many VBP in dogs and cats are probably largely underestimated in SEA.

### 5.2 Prevention and control

As for other diseases, VBD prevention is always better than cure (Dantas-Torres & Otranto, 2016). The prevention of VBD is strongly linked to the control of their arthropod vectors (Otranto, 2018). The control of arthropod vectors such as ticks, fleas, and mosquitoes should be performed simultaneously by using an integrated approach focusing on animals and the environment (Otranto & Wall, 2008). Many acaricides and insecticides in several formulations (e.g. topical sprays, spot-on, baths, dusting powders, and collars), which have a long-lasting effect, and are safe for pets and their owners, are commercially available in SEA. For instance, the combination containing 10% imidacloprid and 50% permethrin can prevent dogs from *Ae. aegypti* mosquito bites for up to 3–4 weeks (Tiawsirisup et al., 2007). The use of afoxolaner can provide an effective treatment (> 96%) against adult *R. sanguineus* (*s.l.*) in dogs in the cases of heavy infestation (Tinkruejeen et al., 2019). Nevertheless, the possible emergence of resistant strains of pathogens and their arthropod vectors due to the indiscriminate use of insecticides, acaricides, and antibiotics should be carefully considered (Otranto et al., 2009). Among the many herbal extracts used as ectoparasites repellents, more than 2,300 plant species were found to have potential mosquito repellent properties across SEA (Tisgratog et al., 2016). For instance, many studies in Thailand revealed the high repellency (up to 8 h) of essential oils extracted from *Angelica sinensis* (dong quai), *Psidium guajava* (guava), *Curcuma longa* (turmeric), and *Piper nigrum* (black pepper) against some mosquito species, such as *Ae. aegypti*, *Ae. albopictus,* and *Cx. quinquefasciatus* (Champakaew et al., 2015; Tawatsin et al., 2006). The essential oil of *Cymbopogon citratus* (lemon grass) has been widely used in many countries in SEA as a cheap and effective mosquito repellent (Shah et al., 2011). Additionally, some biological control agents (i.e. *Mesocyclops* and *Wolbachia*) against *Aedes* spp. have also been applied. The application of *Mesocyclops* for eradication of *Ae. aegypti* in some provinces with high incidence of dengue in Vietnam have achieved a great accomplishment with approximately 90% of *Ae. aegypti* larval populations reduced after one year of intervention (Vu et al., 2005). Along with the MDA, which has been given to people living in LF endemic areas, the control programme of filarial nematodes among reservoir host populations (i.e. domestic dogs and cats) should be considered as one of the comprehensive strategies to archive the target of the GPELF. The use of doxycycline alone or in combination with ivermectin for treatment of *B. malayi* infection in cats has given a good eradication effect to both microfilariae and adult worms (Khowawisetsut et al., 2017). Recently, guidelines for the diagnosis, preventions and treatments of parasitic diseases in companion dogs and cats in the tropics have been prepared by the Tropical Council of Companion Animal Parasites (Dantas-Torres et al., 2020). The guidelines are freely released in multiple languages including English, Bahasa Malaysia, Thai, and Vietnamese (https://www.troccap.com), which are very useful for local veterinarians and pet owners to improve the awareness, prevention and control of VBD.

## 6 Conclusions and research needs

The updated data discussed in this review illustrate a general picture of VBD affecting dogs and cats in SEA, which represent an important issue to animal and public health. Overall, zoonotic VBP, such as *R. felis*, *B. henselae,* and *D. repens*, are of concern to human health in this region. Despite the recently acquired scientific knowledge, many scientific gaps still persist about the eco-epidemiology of the zoonotic VBP, which limit our current understanding and our capability to control them. For instance, although many human cases of dirofilariasis by *D. repens* have been reported (Rohela et al., 2009; Van De et al., 2012), the source of infection as well as the species of mosquito vectors of this nematode in SEA remains unclear. Therefore, the zoonotic transmission cycle of *D. repens* in SEA deserves further investigations. In spite of the acquired data for some countries such as Malaysia and Thailand (Colella et al., 2020; Huggins et al., 2019; Koh et al., 2016; Low et al., 2018; Wongkamchai et al., 2014), data regarding the distribution of the zoonotic VBP in other countries (e.g. Cambodia, Laos, Myanmar, and Timor-Leste) is limited or virtually inexistent. On the other hand, the rapidly changing of environment (e.g. climate change, land use change, and urbanization) in SEA may also alter the distribution and abundance of vectors and VBD (Dantas-Torres, 2015; Lim & Vythilingam, 2013). Therefore, further epidemiological surveillance as well as studies on the impact of those environmental factors to the distribution of the zoonotic VBP in SEA are advocated. Finally, a stronger collaboration between governments, commercial companies, scientists, and medical and veterinary communities should be implemented for a better management of VBD in SEA. The effective control measures of canine and feline VBD as well as their arthropod vectors should be performed simultaneously for a better prevention of zoonotic infections.

## Declaration of competing interests

The authors declare that they have no known competing financial interests or personal relationships that could have appeared to influence the work reported in this paper.

## Authors’ contributions

DO conceived the study. VLN conducted the literature screening, analyzed the data, and wrote the first draft of the manuscript. FDT and DO critically revised the manuscript. All authors read and approved the final manuscript.

## Funding

This study received no specific funding.
